# Combined adsorption and electrochemical oxidation of perfluorooctanoic acid (PFOA) using graphite intercalated compound

**DOI:** 10.1007/s11356-024-32449-0

**Published:** 2024-02-17

**Authors:** Antoine P. Trzcinski, Kouji Harada

**Affiliations:** 1 School of Agriculture and Environmental Science, University of Southern Queensland, West Street, Queensland, 4350 Australia; 2 Department of Health and Environmental Sciences, Graduate School of Medicine, Kyoto University, Kyoto, 606-8501 Japan

**Keywords:** PFOA, Adsorption, Graphite intercalated compound, Electro-chemical oxidation

## Abstract

Perfluorooctanoic acid (PFOA) is a bioaccumulative synthetic chemical containing strong C–F bonds and is one of the most common per- and polyfluoroalkyl substances (PFAS) detected in the environment. Graphite intercalated compound (GIC) flakes were used to adsorb and degrade PFOA through electrochemical oxidation. The adsorption followed the Langmuir model with a loading capacity of 2.6 µg PFOA g^−1^ GIC and a second-order kinetics (3.354 g µg^−1^ min^−1^). 99.4% of PFOA was removed by the process with a half-life of 15 min. When PFOA molecules broke down, they released various by-products, such as short-chain perfluoro carboxylic acids like PFHpA, PFHxA, and PFBA. This breakdown indicates the cleavage of the perfluorocarbon chain and the release of CF_2_ units, suggesting a transformation or degradation of the original compound into these smaller acids. Shorter-chain perfluorinated compounds had slower degradation rates compared to longer-chain ones. Combining these two methods (adsorption and in situ electrochemical oxidation) was found to be advantageous because adsorption can initially concentrate the PFOA molecules, making it easier for the electrochemical process to target and degrade them. The electrochemical process can potentially break down or transform the PFAS compounds into less harmful substances through oxidation or other reactions.

## Introduction

PFAS are a group of man-made chemicals that have been utilized in various industrial and consumer applications due to their unique properties, such as their resistance to heat, water, and oil. PFOA specifically has been used in the production of non-stick cookware, water-repellent clothing, stain-resistant fabrics, and in certain firefighting foams, particularly those used at air bases and airports. PFOA is employed for polymerization in the manufacturing of various fluoropolymers, which have been applied in various industrial and consumer products such as Gore-Tex and Teflon (Mojiri et al. 2023). The widespread use of PFAS in various products and industrial processes has led to their presence in the environment, including water sources, soil, and even in the bodies of animals and humans. Concerns about their persistence, potential health effects, and environmental impact have led to increased scrutiny and efforts to mitigate their presence and use in various applications. Elevated concentrations of PFOA (up to 120 × 10^6^ ng/L), especially in wastewater, can result from industrial discharges, runoff from areas where PFAS-containing products are used, or improper disposal practices (Lei et al. 2022).

The US Environmental Protection Agency (EPA) issued a health advisory level of 70 ng/L for PFOA and PFOS combined in drinking water in 2016 (EPA 2016), but some states require a maximum PFOA level in drinking water at 14 ng L^−1^ (Dadashi Firouzjaei et al. 2022). PFOA was found in Australian drinking water at a concentration of 9.7 ng L^−1^ in 44% of all samples tested in South East Queensland (Thompson et al. 2011), but concentration in groundwater around army bases have been reported at µg L^−1^ levels. For instance, the average aggregate concentration of PFOS (perfluorooctane sulfonate), PFOA (perfluorooctanoic acid), and PFHxS (perfluorohexanesulfonic acid) at an army site in Australia being reported as 4.92 µg/L (micrograms per liter) signifies a notable presence of these per- and polyfluoroalkyl substances (PFAS) in that particular environment (Leung et al. 2022).

Many existing technologies, like polymeric resins (Kothawala et al. 2017), reverse osmosis (Tang et al. 2006), and activated carbon (Szabo et al. 2017) are effective at capturing per- and polyfluoroalkyl substances (PFAS) from water sources. However, these methods do not actually destroy PFAS molecules; instead, they transfer them to another phase or medium, which does present a risk of potential re-pollution if not managed properly. Thompson et al. (2011) even reported that the RO concentrate containing PFAS was released in rivers. Media such as activated carbon or resins must be incinerated or disposed of in landfills (Stoiber et al. 2020). Destruction technologies include sonolysis using ultrasonication (Cheng et al. 2008), microwave (Chou et al. 2013), alkaline hydrolysis (Hao et al. 2021), advanced oxidation processes (Vecitis et al. 2009), and supercritical water oxidation (Pinkard et al. 2021). In the case of treatment at 200 kHz under atmospheric air conditions for 1 h, the removal rate for PFOA was reported as 28%. Similarly, at a lower frequency of 20 kHz but for a longer duration of 6 h, the removal rate for PFAS was recorded at 14.6% (Lei et al. 2020). Microwave is the least effective method for PFOA degradation as 3.1–5% removal efficiency was reported at a power in the range 90–140 W and temperature of 130 °C for 8–12 h (Lee et al. 2009; Chou et al. 2013). Ozonation could remove up to 85% PFOA in aqueous solutions under alkaline conditions (Lin et al. 2012). More recently, photocatalysis was reported to remove up to 46% PFOA in wastewater using zero-valent iron at acidic pH (Xia et al. 2022). Only 42% PFOA could be removed through a hydrogen peroxide process, but 99% was achieved when persulfate was used as a reactant (Bannister 2020). Wang et al. (2019) reported 97% removal after 4 h using a synergic electrochemical process combining cathodic electro-Fenton and anodic oxidation with boron-doped diamond as anode and Fe10MnC as cathode. Electrocoagulation with an air cathode was found to remove 99% PFOA within 45 min at an energy consumption of 0.14 kWh/m^3^ (Mu et al. 2021).

The complete breakdown or cleavage of the carbon–fluorine (C–F) bond in perfluoroalkyl substances (PFAS) is essential to eliminate the risk of re-contamination and potential long-term environmental or health hazards. Some technologies have shown promise in breaking down PFAS, yet they might not always achieve full degradation of all PFAS compounds, leading to the formation of shorter-chain PFAS or transformation products. Even when PFAS are broken down partially, shorter-chain PFAS or other transformation products can still retain some level of toxicity and persistence. These breakdown products might have different environmental behaviors and toxicity profiles compared to their parent compounds, and their impact on ecosystems and human health might not be fully understood. Electrochemical oxidation (EO) has been highlighted in research as a potentially more energy-efficient method compared to sonolysis and ultrasonication for the treatment of perfluoroalkyl substances (PFAS) in water. The energy requirements for EO appear to be considerably lower (ranging from 5 to 132 kWh/m^3^) in comparison to sonolysis (4000–11,000 kWh/m^3^) and ultrasonication (1475 kWh/m^3^). Lower energy consumption could contribute significantly to cost reduction and the feasibility of large-scale application (Sharma et al. 2022). Additionally, EO operates under normal temperature and pressure conditions, which simplifies the operational aspects and lowers infrastructure requirements. Its potential scalability further adds to its attractiveness as a viable treatment option for PFAS-contaminated water sources (Niu et al. 2012). The application of electrochemical oxidation (EO) with low-cost graphene sponge electrodes, as reported by Duinslaeger and Radjenovic (2022), demonstrated promising results in the destruction of C4–C8 perfluoroalkyl substances (PFAS). Their study indicated a range of destruction rates from 16.7 to 67% for PFAS compounds within the C4 to C8 chain length. Using boron-doped diamond (BDD) film electrodes, between 44 and 70% PFOA in wastewater was destroyed at 2.3–21.4 mA cm^−2^ (Uwayezu et al. 2021), while at 75 mA cm^−2^, Pierpaoli et al. (2021) achieved 80% removal of PFOA in landfill leachate.

Using graphite intercalated compound (GIC) as a combined adsorbent and conductive material within an electrochemical system for PFAS removal presents an intriguing method. This method offers continuous regeneration of the adsorbent while applying an electric current, creating a synergistic adsorption and electrochemical oxidation (EO) process. The concept of electrosorption, where an electric field enhances the adsorption capacity of PFAS molecules, has been explored in various studies. Researchers have observed increased removal rates of PFAS, such as PFOA, under the influence of electrical voltage. This enhanced adsorption mechanism primarily involves altering the charge on PFAS molecules, improving their mobility and subsequent adsorption onto the adsorbent material without actual degradation of the PFAS molecules. For instance, Li et al. (2011) reported that 150 times more PFOA were removed when 0.6 V was applied. This electrical voltage increased the charge on PFAS molecules which induces their mobility and improves their adsorption. Their LC–MS work also demonstrated that at 0.6 V no degradation of PFOA took place and that the only removal mechanism was therefore enhanced adsorption in an electrical field. Even though there are numerous studies on the adsorption of PFOA or electrochemical oxidation of PFOA by electrodes, the combined adsorption and electrochemical oxidation of PFOA using GIC has not yet been explored in the literature. The aim of this paper was therefore to quantify the adsorption capacity of GIC toward PFOA and asses its degradation through electrochemical oxidation. The aim was to understand the adsorption mechanisms of PFOA onto GIC and its kinetics of degradation through monitoring of the degradation by-products. Also, the performance of the process was investigated to understand the effect of current density, catholyte solution, and alkalinity on the combined adsorption and electrochemical oxidation of PFOA.

## Methods and materials

### Adsorbent and adsorbate

Expandable graphite intercalation compound (GIC) in the form of flakes was obtained from Sigma-Aldrich (P/N: 808,121). Perfluorooctanoic acid (C_8_F_15_OOH) was purchased from Fluka (P/N: 77,260, ≥ 90% purity). A stock solution containing approximately 10 mg L^−1^ was prepared for the experiments.

### Batch adsorption tests

Batch adsorption experiments evaluate the adsorption capacity of graphite for perfluorooctanoic acid (PFOA). The experiments were carried out in 250-mL Erlenmeyer flasks at a controlled temperature of 25 °C. To conduct the tests, a volume of 100 mL of distilled water containing 1 g of graphite was prepared. Various volumes of a stock solution containing PFOA were added to the flasks to achieve different initial concentrations. These flasks were then placed on an orbital shaker at 150 rpm to facilitate mixing and interaction between the graphite and the PFOA solution. Samples were withdrawn from the flasks at different time intervals to monitor the adsorption process until equilibrium was reached. To analyze the samples, they were filtered using a 0.22-µm filter, likely to separate the graphite and any residual particles from the solution before analyzing the remaining concentration of PFOA. The amount of PFOA adsorbed at equilibrium, *q*_e_ (mg g^−1^), was calculated as

1 $$q\mathrm{e }=\frac{{C}_{o-}{C}_{e}}{M} \times V$$

where *C*_o_ and *C*_e_ (mg L^−1^) are the liquid-phase concentrations for initial sorbate and equilibrium, respectively, *V* is the volume of the solution (L), and *M* is the mass of graphite used. The Langmuir and Freundlich models were used to interpret the adsorption isotherms. The Langmuir model assumes monolayer adsorption on a surface with uniform sites, while the Freundlich model suggests heterogeneous adsorption onto a surface with multiple sites of varying energies (Mohammed et al. 2011). Kinetic models, such as pseudo-first-order and pseudo-second-order, were applied to describe the rate at which the adsorption process occurs over time (Trzcinski et al. 2011).

### Electrochemical reactor

The reactor used to study the combined adsorption and regeneration of graphite is made of Perspex acrylic plastic in a Y-shape. Its technical drawings were reported elsewhere (Mohammed et al. 2012). Details of the experimental set up can be found somewhere else (Trzcinski and Harada 2023). The catholyte compartment is approximately 500 mL containing 3% NaCl and HCl to pH between 1 and 2 (unless mentioned otherwise). About 100 g of graphite was added to fill the regeneration zone (12 cm deep and 5 cm thick). The volume of the reactor chamber is about 6 L, but only 3 L was used for the experiments. The anodic and cathodic compartments were separated by a semipermeable Daramic membrane (Daramic.com, USA). The current was adjusted using a digital power supply (0–3 A, 0–30 V, GPS-3030DD, Instek, Taiwan). First, an air pump was used to keep the graphite in suspension and promote adsorption for 20 min, then the pump was stopped, and the graphite was allowed to settle in the regeneration zone for 2 min, after which the current was switched on for 10 min. For the experiments, three mL of the PFOA stock solution was injected in the 3-L reactor to give an initial concentration of approximately 10 µg L^−1^ in order to simulate typical groundwater contamination found in Australia.

### Liquid–liquid extraction of PFOA

The following procedure outlines the method used for the extraction and analysis of perfluorooctanoic acid (PFOA) from a sample using a series of steps involving extraction, evaporation, and derivatization for analysis via gas chromatography–mass spectrometry (GC–MS).

Preparation of sample and ion-pairing buffer: In a 2-mL Eppendorf centrifuge tube, a 200-µL sample is mixed with 200 µL of an ion-pairing buffer, in this case, tetrabutylammonium hydrogensulfate.

Extraction with methyl tert-butyl ether (MTBE): four hundred microliters of methyl tert-butyl ether (MTBE) is added to extract PFOA. The tube is manually shaken to mix the contents thoroughly and then centrifuged at 5000 rpm for 3 min. After centrifugation, the MTBE layer, which contains the extracted PFOA, is carefully transferred to a glass tube.

Evaporation of MTBE: The MTBE in the glass tube is evaporated at 80 °C under a stream of nitrogen to remove the solvent and concentrate the extracted PFOA.

Addition of internal standard and derivatization: twenty microliters of an internal standard, specifically 1 ng of 11H-perfluoroundecanoic acid, is added to the concentrated sample and evaporated again. This step is performed to aid in quantification and ensure accuracy during analysis.

Derivatization for GC–MS analysis: bis(4-tert-butylphenyl)-iodonium hexafluorophosphate (BtBPI) in acetonitrile (1% w v^−1^) is added (200 µL) to the sample, mixed by pipetting, and then transferred to a GC–MS vial for analysis. Derivatization enhances the volatility and detectability of the analytes in the GC–MS process.

### GC–MS analysis

The analytical setup for the analysis of PFOA using gas chromatography–mass spectrometry (GC–MS) involved a DB-5MS capillary column (30 m long, 0.25 mm internal diameter, and 0.25 µm film thickness) installed in an Agilent 6890 GC-5973MSD instrument. The injection volume for the samples was set at 1 µL as described in Harada et al. (2020). Three identical water samples spiked with perfluorooctanoic acid (PFOA) underwent liquid–liquid extraction and were subsequently injected into the GC–MS for analysis. The analytical results from these samples revealed an average PFOA concentration of 10.92 µg L^-1^ with a standard deviation of 0.68 µg L^-1^.

## Results and discussion

### Adsorption kinetics

**Fig. 1:**
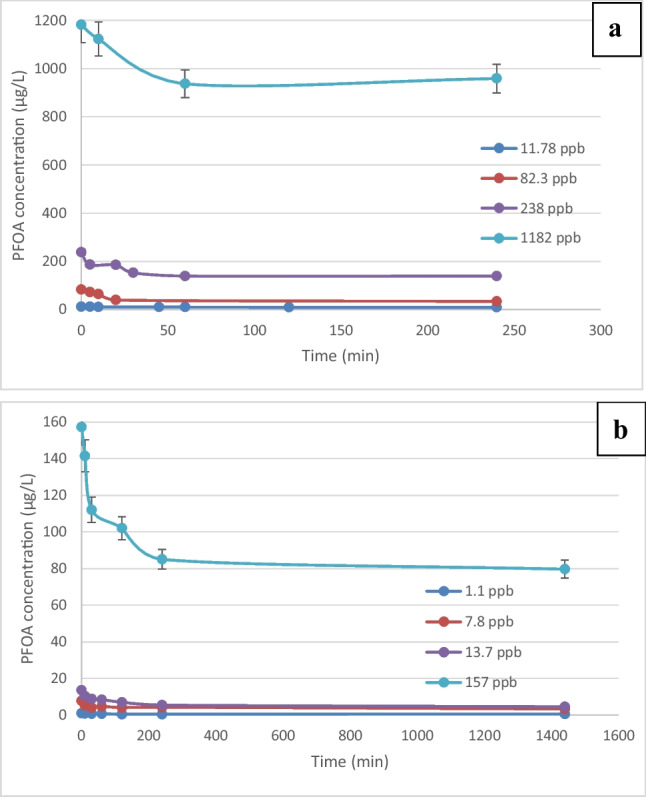
**a** Change in PFOA concentration over a 4-h period using a range of initial concentrations (1182, 238, 82.3, and 11.78 µg L^−1^) during the batch adsorption of PFOA on GIC (10 g L^−1^). **b** Change in PFOA concentration over a 24-h period using a range of initial concentrations (157, 13.7, 7.8, and 1.1 µg L^−1^) during the batch adsorption of PFOA on GIC (30 g L.^−1^). The error bars represent the standard deviation (*n* = 3)

PFOA adsorption kinetics was studied at room temperature (25 °C) in batch experiments at various PFOA concentrations in the range 1.1–1182 µg L^−1^ and two different GIC concentrations (10 and 30 g L^−1^). Figure 1 shows the decrease in concentrations of PFOA due to the adsorption onto graphite and 1–2 h was required to achieve equilibrium at concentrations above 100 µg L^−1^. At lower concentration, equilibrium was reached within an hour. These equilibrium times are relatively longer than for PFOS and other organics which typically require less than 20 min of adsorption on GIC (Brown et al. 2004; Hussain et al. 2015; Flores et al. 2018; Trzcinski and Harada 2023). This is relatively faster that previous adsorption studies on porous activated carbon and anion-exchange resins which can take more than 50 h to reach equilibrium (Du et al. 2014). Removal percentages were in the range 17–41% at 10 g GIC L^−1^ (Fig. 1a) and 43–67% at 30 g GIC L^−1^ (Fig. 1b) which is the maximum GIC concentration in the electrochemical reactor.

**Fig. 2:**
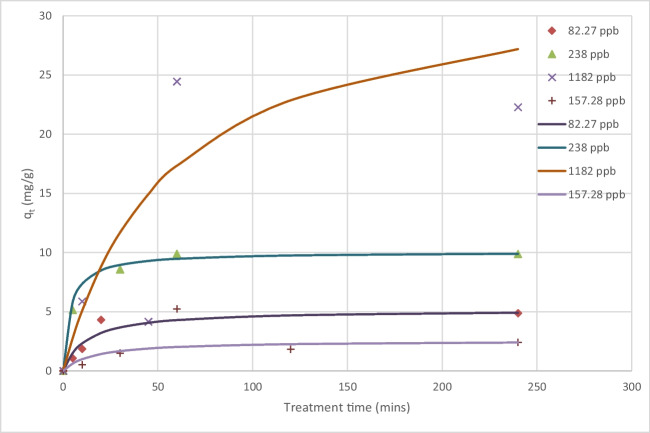
Change in PFOA adsorption capacity during batch adsorption on GIC (data points) and pseudo-second-order kinetic model (curves) for a range of initial concentrations

Figure 2 shows the PFOA adsorption capacity *q* which increased to a maximum of 24.5 µg PFOA g^−1^ GIC after 60 min at an initial PFOA concentration of 1182 µg L^−1^. As the PFOA concentration increased from 1.1 to 1182 µg L^−1^, the adsorption capacity increased from 0.0204 to 22.2 µg g^−1^ (Fig. 2). This is due to the increase in the mass transfer driving force and hence, the rate at which PFOA molecules migrate from the bulk solution to the GIC (Caturla et al. 1998). The maximum adsorption capacity of 24.5 µg g^−1^ GIC is lower than that of PFOS which was found to be 65 µg g^−1^ GIC and this is due to PFOA being less hydrophobic than PFOS because PFOS has one more carbon‑fluorine bond in its perfluoroalkyl chain (Li et al. 2011). This is also consistent with other studies comparing the adsorption of PFOS and PFOA on activated carbons and biochar which exhibit better adsorption capacity toward PFOS (Zhang et al. 2021). High adsorption capacity values have been reported in the literature: 368 mg PFOA/g polyethyleneimine-modified graphene oxide through electrostatic attraction and hydrophobic interaction (Lei et al. 2022), 9.06 mg PFOA g^−1^ zirconium-imprinted magnetic manganese ferrite assembled reduced graphene oxide nanohybrids (Elanchezhiyan et al. 2020), and 35.7 mg PFOA/g granular activated carbon (Zhang et al. 2021). However, some studies have reported lower adsorption capacity in the range 0.025–0.139 µg PFOA g^−1^ biochar amended soil (Askeland et al. 2020).

The calculated values of graphite loading (*qt*) versus time were analyzed for fitting with both pseudo-first-order and pseudo-second-order kinetic models. The results suggest that, although neither model provided a perfect fit, the pseudo-second-order model was deemed better than the first order in describing the adsorption of PFOA onto GIC. In the context of the pseudo-second-order kinetic model, the equilibrium loading values obtained from the experiments (*q*_e,exp_) were found to be closer to the values predicted by the pseudo-second-order model compared to the pseudo-first-order model. Figure 2 displays the curve obtained from fitting the pseudo-second-order model to the experimental data, indicating the agreement between the model and the observed kinetics. Table 1 contains the key kinetic parameters derived from the pseudo-second-order model. These include the kinetic constant (*k*_2_), the calculated equilibrium loading (*q*_e2_), the initial sorption rate, and the coefficient of determination (*R*^2^). The overall conclusion suggests that the pseudo-second-order kinetic model provides a more accurate representation of the adsorption kinetics of PFOA onto GIC in the experimental conditions studied. The model was however not well adapted at the highest concentration of 1181 ppb possibly due to desorption of PFOA at these concentrations because of weak interactions with GIC.

**Table 1:** Parameters for the kinetic models and isotherm coefficients for Langmuir and Freundlich models for the adsorption of PFOA onto GIC

Experimental data		First-order model			Second-order model			
*C*_0_ (µg L^−1^)	*q*_e,exp_ (µg g^−1^)	*k*_1_ (min^−1^)	*q*_e1_ (µg g^−1^)	*R*^2^	*k*_2_ (g µg^−1^ min^−1^)	*q*_e2_ (µg g^−1^)	Initial sorption rate (µg g^−1^ min^−1^)	*R*^2^
11.78	0.2637	0.0359	0.2066	0.6731	0.1636	0.2918	0.0113	0.9603
82.27	4.8763	0.2513	6.1657	0.9149	0.0157	5.1680	0.3743	0.9964
237.74	9.8740	0.1437	8.1319	0.9556	0.0270	10.0503	2.6329	0.9970
1181.81	22.2851	0.0060	19.7174	0.1591	0.0005	33.5570	0.2635	0.3704
1.1	0.0204	0.0242	0.0150	0.4149	1.7227	0.0231	0.0007	0.9379
7.82	0.1174	0.0732	0.0776	0.8311	3.3539	0.1194	0.0462	0.9976
13.7	0.2744	0.0281	0.2074	0.8985	0.1334	0.2994	0.0100	0.9848
157.28	2.4082	0.0253	1.9119	0.8114	0.0244	2.5562	0.1416	0.9040
Langmuir	*K*_L_ (µg g^−1^)	*b*_L_ (L µg^−1^)	*R*^2^	Freundlich	*K*_F_ (µg^1−1/*n*^ L^1/*n*^ g^−1^)	*n*	*R*^2^	
	2.598	0.016	0.9951		0.0442	1.011	0.9351	

### Adsorption isotherms and mechanisms

The coefficient of determination (*R*^2^) in Table 1 suggests that the Langmuir model provided a better fit to the experimental data compared to the Freundlich model. This indicates that the adsorption of PFOA onto the homogeneous GIC followed a monolayer type of adsorption, where there is limited interaction between the adsorbed molecules and neighboring adsorption sites. The Langmuir affinity constant (*b*_L_) of the adsorbent was determined to be 0.016, a value between 0 and 1, suggesting that the sorption process was spontaneous and favorable. This indicates that PFOA molecules were taken up onto the GIC surface with a certain degree of preference or affinity. In the Freundlich model, the value of *n*^−1^ was found to be 0.9, suggesting desirable adsorption characteristics. The Freundlich constant (*K*_F_) value was 0.0442, indicating a relatively low affinity between the sorbents (GIC) and PFOA. The maximum sorption capacity calculated using the Langmuir isotherm model was determined to be 2.598 µg g^-1^. This value is notably lower (three to four orders of magnitude) compared to the sorption capacities achievable using activated carbons, as referenced in the study by Zhang et al. (2021). The adsorption mechanism of perfluorooctanoic acid (PFOA) onto the surface of the graphite intercalation compound (GIC) involves weak interactions rather than a pore-filling mechanism, given the non-porous nature of the GIC surface. The weak adsorption of PFOA onto the adsorbent surface is likely due to van der Waals’ forces or other weak intermolecular forces, as opposed to strong chemical bonding. These forces, such as hydrophobic and electrostatic interactions, facilitate the attachment of PFOA molecules onto the surface of the GIC. During the adsorption process, mechanical stirring might have caused mild particle attrition, leading to the release of weakly adsorbed PFOA molecules from the surface of the GIC particles back into the bulk solution. This phenomenon can contribute to the reversible nature of the adsorption process, where some adsorbed molecules can detach from the surface and re-enter the solution under certain conditions. Overall, the adsorption of PFOA onto GIC appears to occur through weak interactions like hydrophobic and electrostatic forces (Elanchezhiyan et al. 2021), explaining the reversible and relatively weak nature of the adsorption process observed in the study.

The pKa values of PFOA are 0.5–3.8 (Hussain et al. 2022); therefore, the anionic nature of PFOA under acidic conditions allows it to interact through electrostatic forces, facilitating sorption onto surfaces with positive charge sites, such as the GIC surface. However, there are complexities in the association of PFOA molecules with the positive charge sites on the GIC surface. In some cases, the association of PFOA molecules with positively charged sites might be unsaturated due to repulsion behaviors among mobile and immobile PFOA molecules. This repulsion could prevent full coverage of the available positive charge sites on the GIC surface, leaving some active sites unsaturated. Additionally, the hydrophobic tails of closely adsorbed PFOA molecules might interact strongly, forming a structure akin to a hemimicelle. This phenomenon occurs due to the hydrophobic nature of the tails of PFOA molecules, which have a tendency to aggregate and form structures similar to micelles, albeit incomplete due to the surface conditions and limited interaction. These aspects contribute to the complexity of PFOA adsorption onto the GIC surface. The balance between electrostatic interactions, repulsion among PFOA molecules, and the formation of potential hemimicelle-like structures impacts the saturation of available adsorption sites and the overall adsorption behavior of PFOA onto the GIC surface. Understanding these intricate interactions is crucial in elucidating the mechanisms and optimizing the efficiency of PFOA removal processes using materials like GIC.

The critical micelle concentration (CMC) of PFOA, reported to be around 157 mg L^-1^ (Lei et al. 2022), signifies the concentration at which PFOA molecules start to aggregate and form micelles in a solution. For the formation of hemimicelles, the solution concentration should be significantly lower, roughly about 0.01% to 0.001% of the CMC (Du et al. 2014). In the present isotherm study, the initial concentration of PFOA molecules exceeded the concentration required for the critical hemimicelle formation. This suggests that conditions were present for hemimicelle formation over the cationic active sites of the graphite intercalation compound (GIC). The surplus concentration of PFOA molecules might have allowed for the creation of hemimicelle structures at these sites. Moreover, considering the hydrophobic nature of PFOA molecules, bilayer formation could have occurred through tail–tail interactions. This interaction involves the hydrophobic tails of adjacent PFOA molecules attracting each other, leading to the formation of bilayer structures (Hassan et al. 2022), especially when in proximity to surfaces with favorable hydrophobic characteristics, such as the GIC surface. The potential formation of hemimicelles and bilayer structures of PFOA molecules on the GIC surface contributes to the complexity of the adsorption process. These structures might influence the saturation of active sites and affect the overall adsorption behavior of PFOA onto the GIC surface, which is important to consider in understanding the dynamics of PFOA adsorption and its interaction with the adsorbent material.

### Electro-chemical oxidation of PFOA

#### PFOA degradation at low current density

This experiment aimed to study the degradation of PFOA under low current densities, specifically at 2.5 mA/cm^2^. A sample was taken from the spiked water before initiating the adsorption process. The process involved multiple cycles of adsorption and regeneration. During the adsorption phase, PFOA would be captured onto the adsorbent surface by pumping air into the electrochemical reactor. Subsequently, the air pump is turned off to allow the GIC to settle down in the regeneration area where the low current density is applied, intending to induce degradation of the adsorbed PFOA into by-products.

**Fig. 3:**
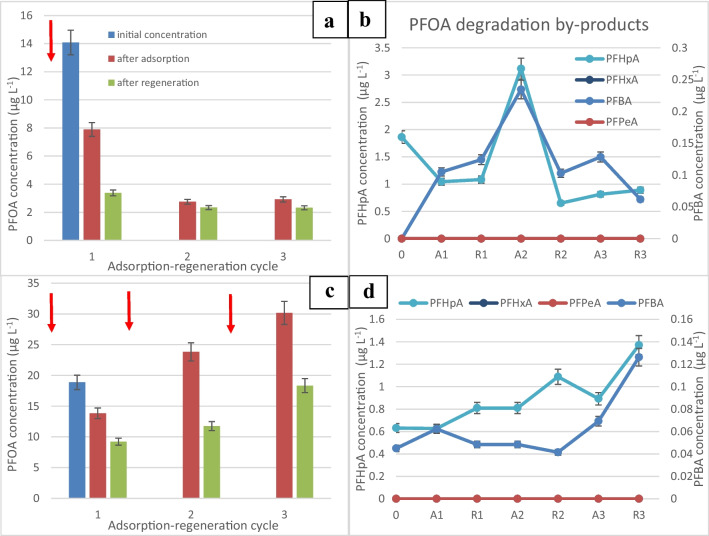
**a** Change in the concentration of PFOA after one spike of concentrated PFOA stock solution into the electrochemical reactor. A, adsorption: 20 min. R, regeneration: 10 min at a current density of 2.5 mA/cm^2^. **b** Change in the concentration of smaller chain perfluoro carboxylic acids after a single PFOA spike at time 0. **c** Change in the concentration of PFOA following multiple spikes. **d** Change in the concentration of smaller chain perfluoro carboxylic acids after multiple PFOA spikes

After the spike at time 0, it can be seen from Fig. 3a that the PFOA concentration decreases from an initial concentration of 14 to less than 3.38 µg/L after the first adsorption/regeneration cycle. The concentration then decreased further to 2.32 µg/L after 3 consecutive cycles achieving 83.5% removal. Figure 3b shows that PFOA was degraded to shorter chain carboxylic acids such as perfluoroheptanoic acid (PFHpA) at concentrations in the range 0.9–3.11 µg/L and perfluorobutanoic acid (PFBA) at lower concentrations (0.061–0.23 µg/L). Perfluorohexanoic acid (PFHxA) and perfluoropentanoic acid (PFPeA) were not detected (< 0.01 µg/L), indicating that they were easily broken down. It has been reported that PFHxA is more water soluble, has less sorption, and has a shorter half-life than PFOA (Shiwaku et al. 2016). After an initial increase during the first regeneration, it can be seen that the concentration of PFHpA and PFBA started to decrease to 0.89 and 0.062 µg/L, respectively, but were not completely removed, indicating that a current density as low as 2.5 mA/cm^2^ could break down PFOA and its by-products to some extent.

In Fig. 3c, the plotted data represents the concentration of PFOA observed in the reactor after each adsorption-regeneration cycle. The experiment involved spiking 3 mL of a PFOA stock solution into the reactor after each cycle. It is clear that PFOA started to accumulate in the reactor as the concentration rose from 13.83 to 30.16 µg/L, but some of the adsorbed PFOA could be broken down as shown by the decrease following each regeneration step. This was accompanied by an accumulation of PFOA by-products, namely, PFHpA and PFBA to 1.37 and 0.126 µg/L, respectively. The accumulation of short-chain perfluorocarboxylic acids indicates that the adsorbed PFOA was broken down into smaller fragments. However, this accumulation of shorter chain compounds occurred after the second cycle, implying that the applied current might not have been adequate to fully oxidize all the PFOA molecules or generate sufficient hydroxyl radicals for their breakdown. Insufficient breakdown of the molecules at a fast enough rate might have led to the saturation of adsorption sites on the graphite. This saturation subsequently caused an increase in the concentration of the breakdown products in the water, as shown in Fig. 3d. This increase signifies that the adsorption sites on the graphite were overwhelmed or reached their capacity due to incomplete breakdown or oxidation of the adsorbed PFOA molecules. This observation highlights the importance of the applied current density in the degradation process. If the current is not sufficiently strong or if the oxidative process is not generating enough reactive species like hydroxyl radicals, the breakdown of the adsorbed PFOA might be incomplete, leading to the accumulation of intermediate breakdown products and the saturation of adsorption sites on the graphite material.

The dominant by-product of PFOA degradation was perfluoroheptanoic acid (PFHpA). Its concentrations were observed to be 10 times higher than those of perfluorobutanoic acid (PFBA), indicating that PFHpA was prominently produced during the breakdown process of PFOA. This observation suggests that PFHpA, despite being a by-product of the breakdown, was still susceptible to both adsorption onto the adsorbent material and subsequent degradation into shorter-chain by-products like PFBA. However, even though PFHpA was the dominant by-product, its presence was notably higher than PFBA, suggesting a more effective breakdown or adsorption of PFHpA compared to PFBA. The lower concentrations and persistence of PFBA in the water, ranging from 0.045 to 0.126 µg/L, imply that PFBA might have been comparatively more challenging to adsorb onto the adsorbent material and break down into smaller by-products than PFHpA. This observation indicates potential differences in the adsorption and breakdown efficiencies among different shorter-chain by-products generated during the degradation of PFOA.

#### PFOA degradation under high current density

**Fig. 4:**
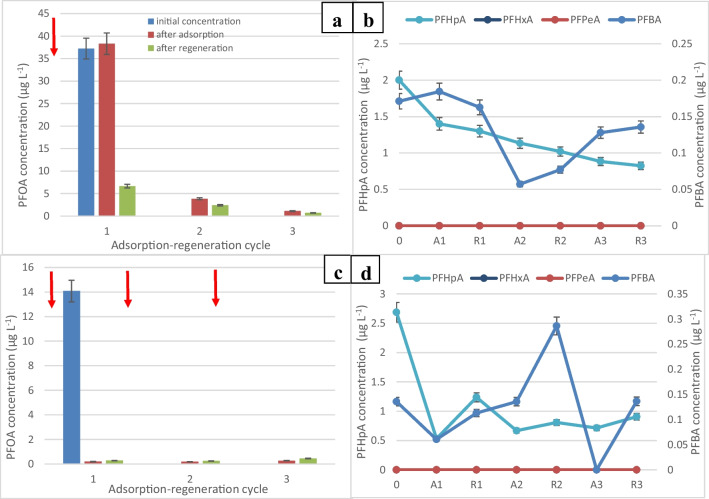
**a** Change in the concentration of PFOA after one spike of concentrated PFOA stock solution into the electrochemical reactor. A, adsorption: 20 min. R, regeneration: 10 min at a current density of 25 mA/cm^2^. **b** Change in the concentration of smaller chain perfluoro carboxylic acids after a single PFOA spike at time 0. **c** Change in the concentration of PFOA following multiple spikes. **d** Change in the concentration of smaller chain perfluoro carboxylic acids after multiple PFOA spikes

Figure 4a shows the degradation of PFOA at a current density of 25 mA/cm^2^ and the PFOA concentration dropped from 38.3 to 6.67 µg/L after the first cycle and down to 0.717 µg/L after 3 cycles, achieving 98% removal. This indicates that the high current density regeneration was oxidizing adsorbed PFOA molecules and was regenerating active adsorption sites on GIC. Similarly, PFHpA concentration decreased from 2 to 0.825 µg/L, while PFBA concentration decreased from 0.171 to 0.136 µg/L.

The higher concentration of perfluoroheptanoic acid (PFHpA) observed during the degradation process implies that the breakdown of PFHpA might have been the limiting factor in the electrochemical oxidation of perfluorooctanoic acid (PFOA) on the graphite intercalation compound (GIC). This suggests that the degradation of PFHpA proceeded at a slower rate compared to other by-products, potentially impacting the overall efficiency of PFOA degradation. The decrease in the concentration of degradation by-products observed in Fig. 4b indicates continuous breakdown of PFOA into shorter-chain perfluorocarboxylic acids and their subsequent adsorption onto the graphite surface or further breakdown. The non-zero initial concentrations of these by-products in the experiment could be due to residual remnants from the previous experiment (as seen in Fig. 3b and d), persisting on the GIC surface, as it might not have been feasible to initiate each experiment with entirely fresh GIC material. Additionally, it is suggested that higher current densities could regenerate the adsorption sites on the GIC due to increased production of hydroxyl radicals (OH^•^) at the anode. This enhanced radical production might contribute to a more efficient breakdown or oxidation of PFOA and its by-products, potentially rejuvenating the adsorption sites and facilitating more effective degradation in subsequent cycles.

Higher current density led to increased electron transfer and acidic environment promoted PFAS degradation and defluorination. After multiple spikes of PFOA, Fig. 4c and d shows 99% removal of PFOA, while Fig. 4d shows 66% destruction of PFHpA, but no significant removal of PFBA remaining around 0.135 µg/L.

The batch experiments conducted revealed that PFOA showed rapid adsorption onto the graphite intercalation compound (GIC) and subsequent breakdown into shorter-chain PFOA by-products. Among these by-products, perfluorohexanoic acid (PFHxA) and perfluoropentanoic acid (PFPeA) were completely removed by the process. However, perfluoroheptanoic acid (PFHpA) and perfluorobutanoic acid (PFBA) could not be entirely eliminated and persisted in the reactor. The percentage removal of these shorter-chain by-products was lower compared to the longer-chain ones. This decrease in removal efficiency with smaller-chain by-products might be attributed to their relatively lower adsorption capacity due to their increased hydrophilicity. Smaller-chain PFOA by-products are typically more hydrophilic compared to their longer-chain counterparts. This increased hydrophilicity can result in reduced affinity for the hydrophobic adsorption sites on the GIC, leading to lower removal efficiency during the adsorption and breakdown process. At low concentration (< 1 µg/L), there is also less driving force for adsorption onto GIC and less chance of hemimicelle formation.

The observations from a water sample taken 12 h after the last cycle reveal that neither PFOA nor its degradation by-products desorbed into the water over time. This suggests that even after the completion of the experimental cycles, these compounds remained adhered to the graphite intercalation compound (GIC) and did not leach back into the surrounding water. Additionally, at the conclusion of the batch experiment, when a sample of the graphite material was taken and subjected to extraction procedures similar to those used for water samples, it was found that both PFOA and its degradation by-products were still adsorbed onto the graphite. This observation implies that as PFOA and its by-products underwent degradation, more adsorption sites on the graphite became available during successive regeneration cycles. Consequently, the decrease in concentration observed in the water samples suggests that PFOA and its by-products remained adsorbed on the graphite surface until they underwent breakdown or degradation. This indicates a dynamic process wherein the adsorbed compounds remained attached to the GIC until they were effectively degraded, without leaching or desorbing back into the water over time.

The proposed process for the adsorption, removal, and degradation of PFOA involves several mechanisms that operate in tandem. Firstly, when GIC immersed in an electrolyte solution is subjected to an external electrostatic field, the particle surfaces acquire either a positive or negative charge (Zhang et al. 2013). This charge on the GIC surfaces leads to an electrostatic attraction between the charged PFOA anions and the oppositely charged GIC flakes, resulting in a phenomenon known as electrosorption. This process combines the adsorption facilitated by the hydrophobic nature of PFOA with electrosorption, effectively enhancing the removal of PFOA onto the GIC surface. This decrease in pH, especially in the absence of alkalinity, occurs due to a combination of factors. Acid diffusion through the membrane and the generation of acid at the anode contribute to this pH drop (Mohammed et al. 2011). The resulting acidic medium creates conditions conducive to the acquisition of a positive charge by PFOA molecules. These positively charged PFOA molecules are then attracted to the negatively charged sides of GIC flakes or the negatively charged electrode.

The electrochemical oxidation process involves the generation of hydroxyl radicals (^•^OH) and electron transfer reactions, playing key roles in the degradation of perfluorooctanoic acid (PFOA). However, it is noted that hydroxyl radicals alone might have limitations in breaking the strong C–F bonds present in PFOA molecules (Ambaye et al. 2022). When the generation of hydrated electrons (e^−^_aq_) is predominant compared to other reactive oxygen species, such as hydroxyl radicals, hydrogen peroxide, ozone, superoxide radicals, and hydronium ions, the degradation of PFOA primarily occurs via a one-electron transfer mechanism due to e^−^_aq_ high reduction potential of − 2.9 V (Deng et al. 2021). This electron transfer results in the conversion of the PFOA anion (C_7_F_15_COO^−^) into radicals (C_7_F_15_COO^•^). Subsequent steps involve Kolbe decarboxylation, radical reactions, intramolecular rearrangements, and hydrolysis, gradually breaking down the PFOA molecules into shorter-chain perfluorocarboxylic acids (PFCAs) (Niu et al. 2012). The degradation of PFOA involves the scission of the chain to release a CF_2_ unit, so C_7_F_15_COO^•^ will react to form CF_2_ and C_6_F_13_COO^•^ which was detected as PFHpA at relatively high concentrations (1–2 µg/L) in this study. In oxygen-rich environments, reaction pathways involve the interaction of C_7_F_15_ with oxygen, leading to the formation of radicals like C_7_F_15_O^•^ and ultimately resulting in the breakdown of PFOA molecules into smaller fragments, including CF_2_O, C_6_F_13_, and CO_2_. In hydroxyl radical–rich environments, C_7_F_15_ can transform into C_7_F_15_OH, leading to the formation of C_7_F_15_O^•^, which follows a similar sequence of reactions resulting in the degradation of PFOA into smaller components, such as C_6_F_13_O^•^ and CF_2_, eventually leading to the production of CO_2_ and HF. C_6_F_13_O^•^ could also recombine with COO^•^ to form PFHpA which was detected in this study. The presence of PFOA by-products in the experiment confirms these proposed degradation pathways. It suggests that hydrated electrons (e^−^_aq_) might also play a role in the process, contributing to defluorination and scission of the centermost C–C bonds in PFOA molecules (Gu et al. 2016). This series of reactions eventually leads to the breakdown and mineralization of PFOA and its by-products into smaller, less persistent compounds and, ultimately, to CO_2_ and HF.

#### Simultaneous adsorption of PFOA and regeneration of GIC

**Fig. 5:**
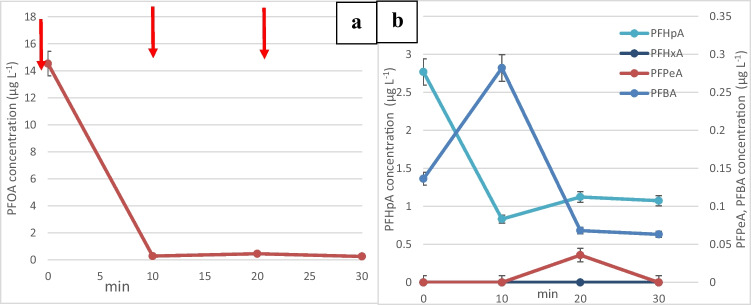
**a** Change in the concentration of PFOA during the simultaneous adsorption and regeneration of GIC following one spike of concentrated PFOA stock solution every 10 min. **b** Change in the concentration of smaller chain perfluoro carboxylic acids during the simultaneous adsorption and regeneration process

Previously, after 20 min of adsorption, the air pump was switched off to allow the GIC to settle down in the regeneration zone to apply the current for 10 min. In this next experiment, the air pump and power supply unit were switched on at the same time to investigate the effect of simultaneous adsorption and regeneration of GIC. Because GIC is kept fluidized by the air bubbles, the current density that can be achieved was significantly lower at 8 mA/cm^2^, but nevertheless, PFOA removal was still 99.4% with 0.262 µg/L after 30 min. It was previously thought that the presence of air bubbles in the regeneration zone would create ohmic resistance which would negatively affect the degradation, but it was found that the simultaneous adsorption and regeneration did not prevent a high PFOA removal as well as a decrease in PFHpA and PFBA concentrations (Fig. 5). As a comparison, Singh et al.’s (2021) study on landfill leachate containing various per- and polyfluoroalkyl substances (PFAS), including perfluorooctanoic acid (PFOA), showcased a lower removal efficiency using an enhanced contact plasma reactor. In their experiments, the application of an applied voltage of 30 kV resulted in 90% removal of PFOA in 10 min.

#### Effect of HCl in catholyte solution

**Fig. 6:**
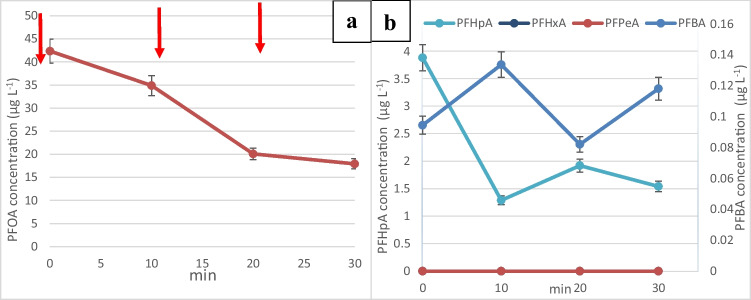
**a** Change in the concentration of PFOA during the simultaneous adsorption and regeneration of GIC following one spike of concentrated PFOA stock solution every 10 min using a catholyte with 3% NaCl without HCl. **b** Change in the concentration of smaller chain perfluoro carboxylic acids during the simultaneous adsorption and regeneration process using a catholyte with 3% NaCl without HCl

The presence of chlorine species in the catholyte solution, which includes sodium chloride (NaCl) and hydrochloric acid (HCl), can contribute to additional indirect oxidation processes during electrochemical treatments. When subjected to an electric field, chloride ions (Cl^−^) can undergo various electrochemical reactions, generating active chlorine species like chlorine gas (Cl_2_), hypochlorous acid (HOCl), and hypochlorite ions (^−^OCl). These species possess strong oxidizing properties and can participate in the degradation of organic compounds, including PFOA, through various pathways. In electrochemical processes involving chloride-containing solutions, these indirect oxidation pathways mediated by electrogenerated active chlorine species can complement the primary degradation mechanisms, potentially enhancing the overall efficiency of pollutant removal or degradation from the aqueous solution. The presence of HCl also provides protons to enhance the adsorption of PFOA on GIC. When HCl was omitted from the catholyte solution (Fig. 6), a maximum current density of only 1 mA/cm^2^ could be applied at 30 V with a pH of 3.5 which resulted in 75% PFOA overall removal. This experiment demonstrated that in order to achieve a high PFOA and by-products removals, a high current density must be applied together with low pH using an acid in the catholyte.

#### Effect of 3% Na_2_SO_4_ + H_2_SO_4_ in catholyte solution

**Fig. 7:**
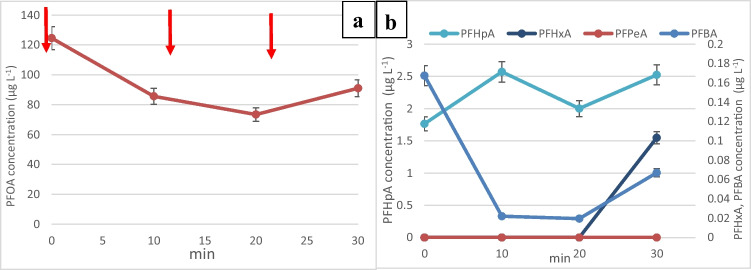
**a** Change in the concentration of PFOA during the simultaneous adsorption and regeneration of GIC following one spike of concentrated PFOA stock solution every 10 min using a catholyte consisting of 3% Na_2_SO_4_ + H_2_SO_4_ (pH < 2). **b** Change in the concentration of smaller chain perfluoro carboxylic acids during the simultaneous adsorption and regeneration process using a catholyte consisting of 3% Na_2_SO_4_ + H_2_SO_4_ (pH < 2)

In order to test the effect of chlorine during the electrochemical oxidation of PFOA, a Cl^−^-free catholyte made of 3% Na_2_SO_4_ and H_2_SO_4_ (pH between 1 and 2) was prepared. Sulfate radical-based methods have garnered attention in environmental remediation due to their notable advantages compared to hydroxyl radicals (^•^OH). Sulfate radicals (SO_4_^•−^) exhibit an equal or higher redox potential, typically ranging between 2.5 to 3.1 V based on activation techniques, compared to hydroxyl radicals (Ghauch and Tuqan 2012). Moreover, sulfate radicals tend to possess higher selectivity and longer half-lives than hydroxyl radicals under diverse conditions. Their increased selectivity means they often target specific pollutants more efficiently, contributing to their effectiveness in degrading various emerging contaminants present in water. As Fig. 7 shows, PFOA removal was low, and its concentration increased after 20 min. PFHxA was even detected at 0.1 µg/L after 20 min, indicating that Cl was essential in the rapid break down of PFHpA to PFHxA and that sulfate radicals were not efficient toward PFOA. Hydroxyl radicals (^•^OH) are highly reactive and often used in advanced oxidation processes due to their ability to oxidize a wide range of pollutants. However, their capacity to break the strong carbon–fluorine (C–F) bonds, commonly found in perfluorinated compounds like PFOA, is limited (Ambaye et al. 2022). Our results indicate that chlorine from the catholyte solution played therefore a significant role in the electrochemical oxidation of PFOA using GIC which was not previously reported. In the absence of chloride, oxidative species such as persulphate and peroxides are formed, and Hussain et al. (2014) reported no effect on the disinfection of *E. coli* in the same process using GIC. In a sulfate radical-based process investigated by Lee et al. (2009), only 12.7% PFOA removal took place in landfill leachate. In the presence of Cl, species such as ClO^−^, Cl_2_, Cl^•^, and HClO are electrogenerated at the anode surface and act through indirect oxidation of PFOA (Mojiri et al. 2023).

#### Effect of alkalinity

**Fig. 8:**
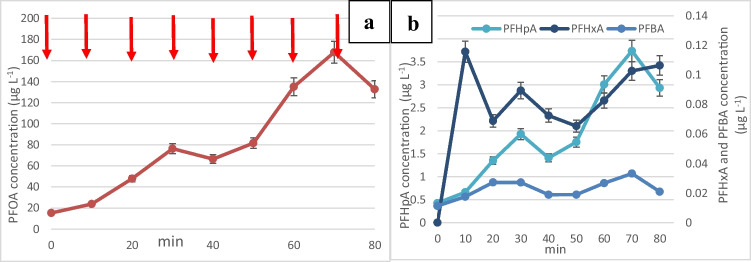
**a** Change in the concentration of PFOA during the simultaneous adsorption and regeneration of GIC following one spike of concentrated PFOA stock solution every 10 min in the presence of 3 g NaHCO_3_/L. Current density was 1 mA/cm^2^ until 40 min and 10 mA/cm^2^ thereafter. **b** Change in the concentration of smaller chain perfluoro carboxylic acids during the simultaneous adsorption and regeneration process in the presence of 3 g NaHCO_3_/L

Previous experiments have highlighted the importance of low pH for enhanced adsorption and electrochemical oxidation of PFOA. However, in real water matrices, alkalinity may be present which may affect the pH in the electrochemical reactor. To investigate the effect of alkalinity, 3 g NaHCO_3_/L (pH ~ 9) was added at the beginning of the experiment and Fig. 8 was obtained. PFOA adsorption and electrochemical oxidation was severely inhibited by alkalinity and PFHpA, PFHxA, and PFBA all increased in concentration not only under 1 mA/cm^2^ (up to 40 min) but also under 10 mA/cm^2^ (after 40 min). The experiment was also repeated with 0.3 g NaHCO_3_/L (pH 8.6) and the results were similar (data not shown), except that PFHxA was not detected, but PFOA, PFHpA, and PFBA increased in concentration. This is because NaHCO_3_ acts as a hydroxyl radical scavenger (Asadi Zeidabadi et al. 2023), which highlights the importance of OH^•^ in the degradation of PFOA in the current study. Future work should include experiments with other scavenger such as methanol for OH^•^, ethanol or Tert butanol for SO_4_^•−^, NO_3_^−^ for e_aq_^−^ (Shang et al. 2022) to better understand the roles of each type of radicals in this process.

The observed data indicates that a higher current density of 25 mA cm^−2^ was significantly more effective in the removal of PFOA and its by-products compared to the lower current density of 2.5 mA cm^−2^. The increased efficiency at the higher current density is attributed to several factors.

- Enhanced electron transfer rates: Higher current densities typically result in increased electron transfer rates, facilitating more rapid and efficient oxidation reactions. This leads to a higher generation of reactive species like hydroxyl radicals (^•^OH) which are crucial in the degradation of pollutants.
- Increased hydroxyl radical production: The elevated current density contributes to greater production of hydroxyl radicals, which possess strong oxidative potential. These radicals play a significant role in breaking down the resilient C–F bonds found in PFOA and its by-products.
- Chlorine-based radical generation: The presence of chloride ions in the electrolyte solution, especially at higher current densities, leads to the generation of chlorine-based radicals (such as Cl_2_^•−^ and HOCl), which are also potent oxidants. These radicals contribute to the degradation of pollutants in the system, complementing the activity of other reactive species.

The combination of these factors at the higher current density of 25 mA cm^−2^ resulted in increased efficiency in the removal of PFOA, perfluoroheptanoic acid (PFHpA), and perfluorobutanoic acid (PFBA), showcasing higher removal percentages across the board compared to the lower current density. In this experiment, the reaction kinetics followed a first order (*R*^2^ = 0.9964) for PFOA with a rate constant *k*_1_ = 0.0449 min^−1^ and a half-life of 15.4 min which is very similar to the kinetics of PFOS removal that we obtained in the same process (Trzcinski and Harada 2023). On the other hand, PFHpA followed a second-order kinetics (*R*^2^ = 0.9969) with rate constant *k*_2_ = 0.008 L/µg.min, half-life of 62.4 min. In agreement with previous results, the degradation rate for PFOA is faster than their by-products (PFHpA and PFBA) (Niu et al. 2012). The study conducted by Niu et al. (2012) employing a Ce-doped modified porous nanocrystalline PbO_2_ film electrode demonstrated a 96.7% removal rate of PFOA. The observed kinetics in their study, which followed a first-order reaction with a rate constant of 3.7 × 10^−2^ min^−1^ and a half-life (*t*_1/2_) of 18.7 min, bear similarities to the results obtained using the graphite intercalation compound (GIC) in our study.

Electrochemical oxidation has often demonstrated higher efficiency in degrading perfluorooctane sulfonate (PFOS) compared to PFOA. PFOS typically contains the sulfonate group and exhibits a greater susceptibility to electrochemical degradation due to the easier breakage of the carbon–sulfur (C–S) bond compared to the carbon–fluorine (C–F) bond in PFOA (Leung et al. 2022). On the other hand, advanced oxidation/reduction processes (AOP/ARP) activated with various means like UV (ultraviolet), ultrasound, or ionizing radiation have shown higher degradation efficiencies toward PFOA. These methods, which generate highly reactive species such as hydroxyl radicals (^•^OH) or sulfate radicals (SO_4_^•−^), are effective in attacking and breaking down the robust C–F bonds present in PFOA molecules. However, our results showed that the novel electrochemical reactor was as efficient toward PFOA compared to PFOS in terms of removal percentage and kinetics (Trzcinski and Harada 2023).

Electrochemical oxidation of PFOA demonstrates a notably lower energy requirement compared to various other technologies like ultrasonication. The energy demand for electrochemical oxidation appears to be substantially more efficient, accounting for only 5.85 kWh per cubic meter of treated water over a 30-min treatment period, compared to 1475 kWh m^−3^ water treated for ultrasonication as highlighted in Sharma et al. (2022) research. This method’s efficiency is further bolstered by its operational advantages: it does not necessitate high temperature, extreme pressure, or the use of harsh chemicals. Instead, it relies on the generation of hydroxyl radicals and the in situ regeneration of the adsorbent, which contribute significantly to the degradation process. However, an interesting point to note is the dependence of the process’s efficiency on pH levels. As indicated by Leung et al. (2022) research, the degradation efficacy of PFOA is notably higher at lower pH levels. This observation aligns with the increased generation and activity of reactive species like hydroxyl radicals under acidic conditions, optimizing the degradation process. The significantly lower energy consumption, coupled with the process’s ability to operate under mild conditions while achieving efficient degradation, positions electrochemical oxidation as a promising and environmentally favorable method for addressing PFOA contamination in water treatment applications.

The adsorption/regeneration process demonstrates the ability to bring PFOA concentrations below health-based guideline values (10 µg L^−1^) set by the Australian government, particularly in recreational waters (NHMRC 2022). Achieving levels below 1 µg L^−1^ after three cycles is a positive outcome and indicates its potential for remediation in certain environmental settings. However, reaching the stricter health-based guideline value of 0.07 µg L^−1^ for drinking water might be more challenging due to the stringent requirements. The process’s efficacy in removing PFOA is commendable, but it is apparent that addressing smaller compounds like perfluorobutanoic acid (PFBA) might require an adsorbent material with a higher affinity specifically tailored to effectively capture and degrade these smaller-sized contaminants. This insight into the process’s performance against different PFAS compounds, along with the comparison to health-based guidelines, provides valuable information for its potential application. While it might not yet meet the stringent requirements for drinking water, its efficiency in reducing concentrations to below the recreational water guidelines showcases its potential for targeted environmental remediation purposes. Continuing research to optimize the process for a broader range of PFAS compounds or developing tailored adsorbents could enhance its effectiveness for diverse water treatment applications.

## Conclusions

The adsorption of PFOA onto GIC occurs rapidly within a relatively short timeframe of 2 h. However, the efficiency of adsorption alone appears to be somewhat limited, as only 43% to 67% of the PFOA was removed through adsorption. The maximum adsorption capacity determined from the Langmuir isotherm was calculated to be 2.598 µg g^−1^. PFOA was readily broken down following a first-order kinetics (*k* = 0.0449 min^−1^ and *t*_1/2_ = 15.4 min), achieving 99% removal down to 717 ng L^−1^ using a current density of 25 mA/cm^2^ after 3 cycles of 20 min adsorption followed by 10 min of electrochemical oxidation. During simultaneous adsorption and regeneration, the process achieved 99.4% PFOA removal down to 0.262 µg/L after 30 min at 10 mA/cm^2^. Breakdown by-products included short-chain perfluoro carboxylic acids such as PFHpA, PFHxA, PFBA. PFHpA, and PFBA could be broken down but required a higher current density of 25 mA/cm^2^ achieving 66% and 21% removal, respectively. The rapid removal of PFOA to levels below 1 µg L^−1^ within a short duration of 10 min and using an energy consumption of less than 2 kWh m^−3^ represents promising progress in PFOA remediation. However, the challenge lies in achieving complete removal, particularly of the short-chain by-products that might persist in the treated water. This hybrid approach may offer benefits such as increased efficiency in removing PFAS from water, potential treatment of a wider range of PFAS compounds, and the possibility of reducing the volume of waste generated compared to individual treatment methods.

## Data Availability

Data are available on request.
